# Short-term learning effect of ChatGPT on pharmacy students' learning

**DOI:** 10.1016/j.rcsop.2024.100478

**Published:** 2024-07-23

**Authors:** Kristian Svendsen, Mohsen Askar, Danial Umer, Kjell H. Halvorsen

**Affiliations:** Department of Pharmacy, Faculty of Health Sciences, UiT – The Arctic University of Norway, Tromsø, Norway

**Keywords:** ChatGPT, Large language modelling, Pharmacy education, Experimental study design

## Abstract

**Introduction:**

Students in pharmacy are positive towards integrating artificial intelligence and ChatGPT into their practice. The aim of this study was to investigate the direct short-term learning effect of using Chat GPT by pharmacy students.

**Methods:**

This was an experimental randomized study. Students were allocated into two groups; the intervention group (*n* = 15) used all study tools and ChatGPT, while the control group (*n* = 16) used all study tools, except ChatGPT. Differences between groups was measured by how well they performed on a knowledge test before and after a short study period.

**Results:**

No significant difference was found between the intervention and control groups in level of competence in the pretest score (*p* *=* *0.28*). There was also no significant effect of using ChatGPT, with a mean adjusted difference of 0.5 points on a 12-point scale. However there was a trend towards a higher proportion of ChatGPT participants having a large (at least four point) increase in score (4 out of 15) *vs* control group (1 out of 16).

**Conclusion:**

There is a potential for positive effects of ChatGPT on learning outcomes in pharmacy students, however the current study was underpowered to measure a statistically significant effect of ChatGPT on short term learning.

## Highlights

• ChatGPT has potential as an educational tool for pharmacy students.
• Pharmacy students who use ChatGPT showed an improvement in test scores in an experimental study setting.
• Studies that investigate the effect of ChatGPT as an educational tool on long-term learning are warranted.
• Pretest scores were positively associated with improvement in test scores.

## 1 Introduction

Artificial Intelligence has been used in teaching future health care professionals in communication skills by being virtual patients and physicians.^1^ In addition, AI chatbots have shown positive effects on learning outcomes for students in general.^2^ These studies have all been conducted on older AI/chatbot tools while students since November 2022 have had the chance to use generative AI tools such as the Chat Generative Pre-trained Transformer (Chat GPT). The introduction of ChatGPT at universities has been considered as a paradigm shift.^3^ The research community has begun to untangle how well this tool can provide information in pharmacy related topics. In one study it was shown that ChatGPT can supply answers to medicine therapy management cases^4^ and in another study that it can provide answers to questions related to the treatment of hypothyroidism in pregnancy fairly well.^5^ Moreover, a recent study reported that ChatGPT produces accurate answers to medical queries as judged by academic physician specialists, although the answers had some important limitations.^6^ Finally, a study from Thailand showed that ChatGPT performed worse than pharmacy students in an exam in the pharmacotherapy of shock and electrolyte disorders.^7^

Students in pharmacy are positive towards integrating artificial intelligence (AI) and ChatGPT into their practice and indicate a significant interest in expanding their education on AI, while at the same time having a lack of knowledge about these tools.^8^^,^^9^ Recently a guide to the use of AI for pharmacy students was published.^10^

The introduction of large language model (LLM) tools such as ChatGPT thus requires more research into how it can be used by students, what the effect of using such tools can be for learning outcomes given that simpler chatbots have shown a positive effect.^2^ The aim of our study was to investigate the direct effect of using Chat GPT on short term learning in pharmacy students using an experimental study design.

## 2 Material and methods

A total of 31 pharmacy students agreed to participate in an experimental randomized study that allocated pharmacy students into two groups; the intervention group (*n* = 15) were allowed to use all study tools and ChatGPT (ChatGPT 4.0 provided as a privacy enhanced version from the university named ChatUIT), while the control group (*n* = 16) could use all study tools, except ChatGPT. Before randomization, students were stratified on study year to ensure that study year was balanced. Participants were from both the three year Bachelor of Pharmacy (B.Pharm) program and two year Master of Pharmacy (M.Pharm) program. The study was conducted in February of 2024.

A sample size calculation was conducted while planning the study. In total there were around 100–120 B.Pharm and 50 M.pharm students available and the study should have had around 80 participants to be able to detect a statistical significant effect. Unfortunately the participation rate was lower than expected even if students informed about the study both online and physically in lectures.

The effect was assessed by administering a knowledge test covering the pharmacy curriculum, before and after the learning period. This test was developed by getting lecturers to create questions and answers related to their subjects. The exact same test was given before and after the learning period, but the participants did not know this in advance and was simply told to use the pre-test as input into what they should spend time learning. They were not told the correct answers and the test was not available for them during the learning period. Students were monitored during the study and test periods to ensure that only ChatGPT participants used the tool and that no participants conducted the tests in an unreasonable short time.

**Fig. 1:**
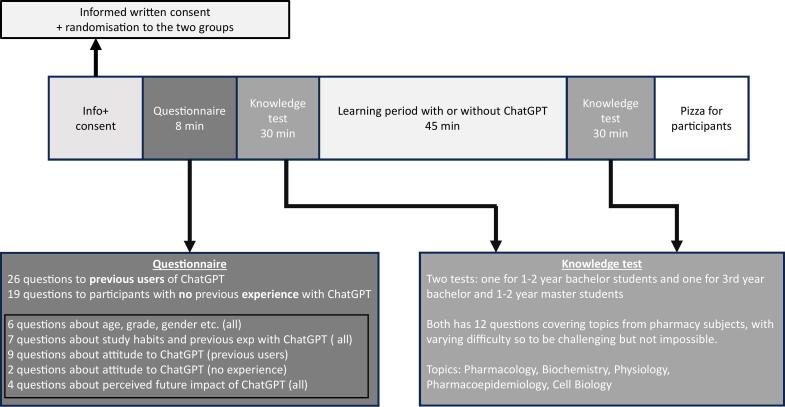
Describe the flow of the study, including information about the questionnaire and the knowledge test, from the student arrives at the lecture theatre, until the study ends (about 3 h in total).

In addition to the knowledge test, the students were also given a questionnaire designed to assess their attitude and knowledge about ChatGPT, as well as questions related to study habits and how they performed on the last exam. Questions in the questionnaire were partially adapted from a study from Hasan et al.^9^ See supplementary 1 for the full questionnaire The set up of the study is shown in Fig. 1.

An independent *t*-test was conducted to investigate group differences between pre-test and post-test scores. An ANCOVA test was conducted to assess the difference between the groups post-test scores while controlling for the pre-test scores. Finally, the balance of variables between the intervention and control group was assessed, and variables with an imbalance were assessed as potential confounders by using a directed acyclic graph diagram, see supplementary 2. Variables found to confounders were included in a linear regression analysis quantifying the effect of ChatGPT on the final knowledge test score. Test of regression assumptions are reported in supplementary 4.

## 3 Results

### 3.1 Participants' characteristics

The participants' characteristics are displayed in Table 1. Of 31 students, 2/3 were females and the participants median age were 23. Fourteen students attended the bachelor program, while 17 attended the master program.

**Table 1:** Description of the demographic data of the participating pharmacy students.

Variable	Variable categories	Total (*N* = 31)	GPT group (*N* = 15)	Control group (N = 16)	*P*-Value
Gender, n (%)	Not answered	1 (3.2)	1 (6.7)	0	0.557
	Male	10 (32.3)	5 (33.3)	5 (31.2)	
	Female	20 (64.5)	9 (60.0)	11 (68.8)	
Age, mean (SD)		25.3 (5.8)	24.3 (4.6)	26.2 (6.8)	0.378
Study year, n (%)	1. yr. B.Pharm	6 (19.4)	3 (20.0)	3 (18.8)	0.899
	3. yr. B.Pharm	8 (25.8)	4 (26.7)	4 (25.0)	
	1. yr. M.Pharm	14 (45.2)	6 (40.0)	8 (50.0)	
	2. yr. M.Pharm	3 (9.7)	2 (13.3)	1 (6.2)	
Last Grade given (A-F, F is fail), n (%)	A	2 (6.5)	1 (6.7)	1 (6.2)	0.248
	B	12 (38.7)	8 (53.3)	4 (25.0)	
	C	7 (22.6)	4 (26.7)	3 (18.8)	
	D	4 (12.9)	0	4 (25.0)	
	E	1 (3.2)	0	1 (6.2)	
	Not answered	5 (16.1)	2 (13.3)	3 (18.8)	
Experience using ChatGPT, n (%)		21 (67.7)	11 (73.3)	10 (62.5)	0.704
Computer normally used, n (%)	Mac	12 (38.7)	6 (40.0)	6 (37.5)	0.363
	Windows	17 (54.8)	9 (60.0)	8 (50.0)	
	iPad\tablet	2 (6.5)		2 (12.5)	
Ability to organize own studies, n (%)	Very Good	3 (9.7)	2 (13.3)	1 (6.2)	0.07
	Good	13 (41.9)	9 (60.0)	4 (25.0)	
	Sufficient	11 (35.5)	4 (25.0)	7 (43.8)	
	Bad	4 (12.9)	0	4 (25.0)	
Study time (total time spent per day), n (%)	1–2 h	8 (25.8)	4 (26.7)	4 (25.0)	0.56
	2–4 h	10 (32.3)	5 (33.3)	5 (31.2)	
	4–6 h	11 (35.5)	6 (40.0)	5 (31.2)	
	>6 h	2 (6.5)	0	2 (12.5)	
Lectures participation, n (%)	Always	19 (61.3)	8 (53.3)	11 (68.8)	0.323
	Almost always	11 (35.5)	7 (46.7)	4 (25.0)	
	Rarely	1 (3.2)	0	1 (6.2)	
Pre-test score, mean (SD)		5.0 (2.0)	5.4 (2.0)	4.6 (1.9)	0.281
Post-test score, mean (SD)		6.8 (2.4)	7.7 (2.0)	5.9 (2.5)	0.041*
Pre-test response time, mean (SD)		20.3 (5.1)	20.8 (5.8)	19.7 (4.5)	0.559
Post-test response time, mean (SD)		9.2 (3.2)	8.5 (3.6)	9.9 (2.8)	0.236

**n** = number of participants, **SD** = standard deviation.

### 3.2 Knowledge test

There was no significant difference between the intervention and control groups in level of competence in the pretest score (*p* *=* *0.28*). However, there was a significant unadjusted difference in the post-test score (*p* *=* *0.04*). When adjusting for the pre-test score (ANCOVA), the difference in post-test scores did not remain significant (*F* *=* *3.19, p* *=* *0.085*). Furthermore, a Linear regression model was applied adjusting for pre-test scores, difference in response between pre- and post-tests for the same participant, last study grade, and age centered around the median age (23 years). In this model intervention participants scored a non-significant 0.521 points higher on their post-test than the control one (p-value 0.55). The results of the full model are included in supplementary 3.

**Fig. 2:**
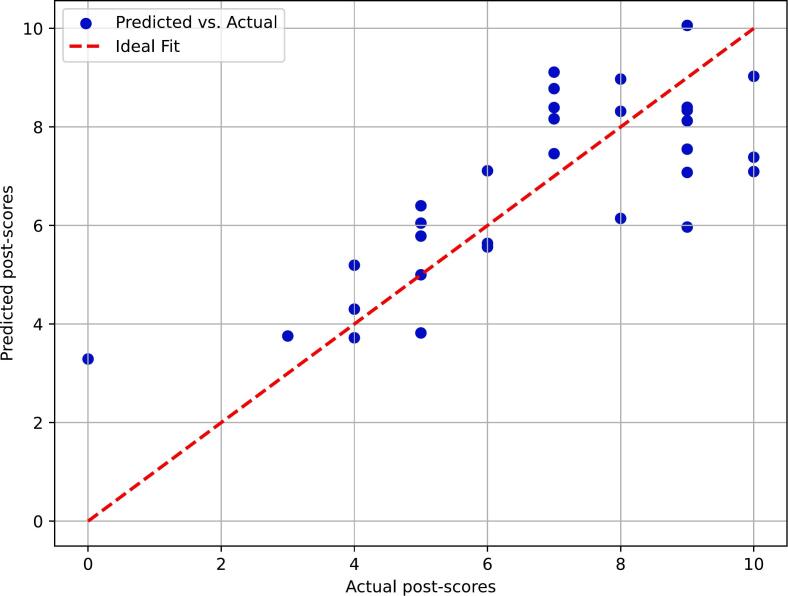
A scatter plot displaying the actual vs predicted post-test scores from the regression model. The red line represents the ideal fit if the predicted values perfectly match the actual values.

We assessed the quality of the model by plotting the predicted and actual score as shown in Fig. 2. The figure shows there is fairly good alignment of predicted and actual scores.

**Fig. 3:**
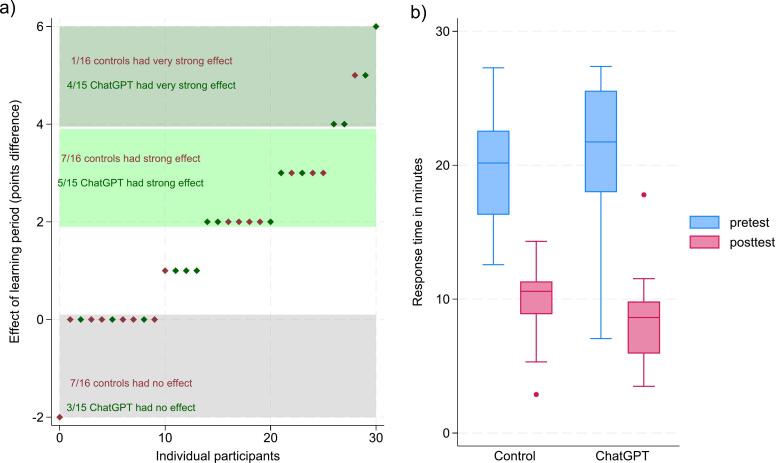
Individual effect stratified by intervention (dark green) and control (maroon) (a). Boxplot with response time in minutes for pre-test and t post-test, stratified by controls and intervention (b). (For interpretation of the references to colour in this figure legend, the reader is referred to the web version of this article.)

More students in the intervention group had a strong or very strong effect of the learning period. 9 out of 15 had an increase of at least 2 points and 4 out of 15 had at least 4 points increase. In the control group, 8 out of 16 had a 2-point increase and only 1 out of 16 had at least 4 points increase (Fig. 3a). There was a larger reduction in time spent on the post-test as compared with pre-test with 12.3 min on average in the ChatGPT group and 9.8 min in the control group (see Fig. 3b).

### 3.3 Knowledge and attitude questionnaire

Participants generally knew about ChatGPT (97% had heard about the tool) and 21 out of 31 participants had previous experience using it. Of these 21 participants 67% would recommend other students to use it for studying. In general participants were aware of advantages and challenges using ChatGPT.

## 4 Discussion

While our study could not detect a statistically significant difference in learning when using ChatGPT, it showed a trend towards a greater increase in test scores and a greater decrease in time spent to answer the knowledge test. The unadjusted difference in test score increase was one whole point for the ChatGPT group which corresponds to around 8% in a test using percentages. The linear regression adjusted results were around 0.5 points, still a relevant difference, even if our study cannot rule out that this difference could be coincidental. When we looked at participants with a strong or very strong effect, a larger proportion of ChatGPT participants had this effect and especially in participants with an at least four points increase.

This study quantifies the potential of using ChatGPT when doing short-term studying in pharmacy subjects. Previous studies have shown that ChatGPT can provide relevant and correct information in pharmacotherapy and in solving clinical cases^4^^,^^5^. However, these studies could not discern the effect of adding ChatGPT to a mix of study tools and techniques. It has been shown that chatbots can have a positive effect on teaching some types of topics such as communication skills^1^^,^^2^, but our study is the first to look at the general effects of ChatGPT on learning more widely for pharmacy students. The study was not powered to be able to see an effect in different topics, but it provides a starting point for further research into how LLM tools can be used in pharmacy education.

For future research it is important to bear in mind that tools like ChatGPT are constantly evolving and improving. As an example, in a study from Thailand in early 2023 it was found that pharmacy students were better at pharmacotherapy than ChatGPT. This might not be true now in 2024 and will almost certainly not hold true in the future as the AI tools get better and can search the internet. In addition, it is important to consider students' evolving experience with using such tools. Students have been shown to be generally positive to such tools^8^^,^^9^ and that was also true in our participants, however even 1.5 years after the introduction only 21 out of 31 participants had tried ChatGPT before, and it will likely be important for schools and universities to teach the general basic use of tools like ChatGPT and to provide specific guidance to how it can be used in a good way in each topic/field/study program. While professions such as psychology has started discussing how ChatGPT can be taught to students^11^ A literature review has looked at advantages and disadvantages to using ChatGPT in teaching students.^12^ While they list many benefits, studies have reported risks such as overdependence on these tools and issues with academic integrity. This means that Schools of Pharmacy also need to focus on teaching students to use LLM tools in a way where the benefits outweigh the potential risks.

There are some limitations to our research, firstly the study was not designed to measure if knowledge is retained over the longer term; this is difficult in a controlled study setting, and other studies with different designs should be conducted in this setting. However, it is reasonable to assume that similar effects in long term learning should be possible if students use the tool in reasonable way. Secondly this was a small study with only pharmacy students from one institution. It did not reach the required number of participants to statistically detect the effect of ChatGPT and the generalizability of the results will be limited since students in other countries and institutions might have a different starting point regarding knowledge, skills, and competencies in using AI tools such as ChatGPT.

However, the study used a robust experimental design that allows to isolate the effect of ChatGPT in a way that observational study designs cannot do, and it was felt that getting timely results was essential and that meant that it was not possible to do a multicenter study within the timeframe of running the study during the first months of 2024. The results were analyzed using a multivariable linear regression where variables were selected using a directed acyclic graph and by observing group differences. Even if, there could be additional confounders, using only our included variables the model showed a good model fit and predicted test scores were mostly close to actual test scores.

All in all, our results show a potential beneficial effect of using ChatGPT. We believe that future studies should aim to test different ways of using tools like ChatGPT so that pharmacy educators can develop optimal ways to teach students the best ways of using these tools. We believe that we as a profession should be bold and embrace new tools both in education and in our professional life and we hope that our study has provided a tiny piece of the enormous puzzle that is how LLMs can be used in the best possible ways moving forward.

## 5 Conclusion

This study was not powered to measure a statistically significant effect of ChatGPT on short term learning, but the results indicate that there might be a potential for positive effects of ChatGPT on learning outcomes in pharmacy students. There is need for larger multicenter studies of the effect of tools like ChatGPT on learning outcomes and how pharmacy students use these tools.

## Funding

This research did not receive any specific grant from funding agencies in the public, commercial, or not-for-profit sectors.

## CRediT authorship contribution statement

**Kristian Svendsen:** Writing – review & editing, Writing – original draft, Supervision, Project administration, Formal analysis, Conceptualization. **Mohsen Askar:** Writing – review & editing, Formal analysis, Data curation, Conceptualization. **Danial Umer:** Writing – review & editing, Formal analysis, Data curation, Conceptualization. **Kjell H. Halvorsen:** Writing – review & editing, Writing – original draft, Supervision, Conceptualization.

## Declaration of competing interest

The authors declare that they have no known competing financial interests or personal relationships that could have appeared to influence the work reported in this paper.

## Appendix A Supplementary data

Supplementary material 1 Image 1

Supplementary material 2 Image 2

Supplementary material 3 Image 3

Supplementary material 4 Image 4
