# Supplementary material for: Short-term learning effect of ChatGPT on pharmacy students' learning
Source: Explor Res Clin Soc Pharm. 2024 Jul 23;15:100478. doi: 10.1016/j.rcsop.2024.100478 (PMC11321390; doi:10.1016/j.rcsop.2024.100478)
Supplement: Supplementary file 1 — Supplementary material 1 [file mmc1.docx]

## Appendix A: Questionnaire about knowledge and attitudes to ChatGPT

*Table A1: Questions and options for answers for questionnaire*

| **Q. No.** | **Question** | **Options** |
| --- | --- | --- |
| 1 | Your assigned student number | N.A. |
| 2 | What is your gender? | Male |
|  |  | Female |
|  |  | Other |
|  |  | Prefer not to say |
| 3 | How old are you? | Free text number |
| 4 | What year of study are you in? | First year |
|  |  | Second year |
|  |  | Third year |
|  |  | First year of master's |
|  |  | Second year of master's |
| 5 | What is your most recent study grade? (Choose the last grade you received that was a letter grade) | A-F |
|  |  | Don’t want to answer |
| 6 | What type of computer are you currently using for your studies? | Mac |
|  |  | Windows |
|  |  | Chromebook |
|  |  | iPad/tablet |
|  |  | Other tablet with keyboard |
| 7 | Where do you usually study? | Library |
|  |  | Home |
|  |  | Reading Room |
|  |  | Other |
|  | If other, please specify. (This question only appears if "Other" is selected in question (7)) |  |
| 8 | How well are you able to organize your own study schedule? | Very Bad |
|  |  | Bad |
|  |  | Sufficient |
|  |  | Good |
|  |  | Very Good |
| 9 | How many hours per day do you typically spend studying? Time you spend on studies beyond mandatory teaching. | Less than 1 hour |
|  |  | 1-2 hours |
|  |  | 2-4 hours |
|  |  | 4-6 hours |
|  |  | More than 6 hours |
| 10 | How often do you attend scheduled teaching sessions? e.g., lectures, labs, seminars, etc. | Always |
|  |  | Almost always |
|  |  | Often |
|  |  | Rarely |
| 11(a) | What study techniques do you prefer?  Summarizing notes, Group studies, Active reading, Flashcards, Digital resources  (Note: Each study technique has all the four options mentioned next column) | Always |
|  |  | Often |
|  |  | Rarely |
|  |  | Never |
| 11(b) | If you use other study techniques, please describe them. |  |
| 12 | Have you heard of ChatGPT? | Yes/No |
| 13 | Do you use ChatGPT in your studies? | Yes/No |
| 14 | Which version of ChatGPT do you use? | ChatGPT 3.5 (Free version) |
|  |  | ChatGPT 4 (Subscription version) |
| 15 | ChatGPT can help me understand complex concepts. How much do you agree with this statement? | Likert Scale § |
| 16 | ChatGPT is a valuable tool for enhancing my learning in pharmaceutical subjects. How much do you agree with this statement? | Likert Scale § |
| 17 | I can trust the answers from ChatGPT. How much do you agree with this statement? | Likert Scale § |
| 18 | What do you think are the advantages of using ChatGPT for studying pharmaceutical subjects? | Answers directly to questions |
|  |  | Answers tailored to my level |
|  |  | Can have a conversation with the bot |
|  |  | Time saving |
|  |  | Other advantages |
|  | If other advantages, please comment. This question only appears if "Other advantages" is selected in question (18). |  |
| 19 | What disadvantages do you associate with using ChatGPT for your studies? | Struggles with complex tasks |
|  |  | Vulnerable to biases |
|  |  | Dependent on internet connection |
|  |  | Misinterpretation |
|  |  | Other disadvantages |
|  | If other disadvantages, please comment. This question only appears if "Other disadvantages" is selected in question (19). |  |
| 20 | Have you had any frustrating experiences while using ChatGPT for your pharmacy studies? | Yes/No |
|  | If yes, please describe. This question only appears if "Yes" is selected in question (20). | Free text |
| 21 | I recommend ChatGPT to other students in an academic context. How much do you agree with this statement? | Likert Scale § |
| 22 | Do you understand how ChatGPT works? (the technology behind ChatGPT) | Know nothing about it |
|  |  | Know a little about it |
|  |  | Know a good deal about it |
|  |  | Know a lot about it |
| 23 | Why do you not use ChatGPT? | Not relevant to my studies |
|  |  | I haven't heard about it |
|  |  | It provides false information |
|  |  | I feel I don't need it |
|  |  | Haven't gotten around to it/haven't had the time |
|  |  | Other |
|  | If other, please comment. This question only appears if "Other" is selected in question (23). |  |
| 24 | Will you use ChatGPT in your studies in the future? | Yes/No |
|  |  | Don’t Know |
| 25 | What do you think is ChatGPT's impact on life in the future? | Negative |
|  |  | Positive |
|  |  | Not Sure |
| 26 | What could be the biggest problem if we are allowed to use ChatGPT in exams in the future? Choose the option that you think is most important | Exams become meaningless |
|  |  | Students learn much less |
|  |  | It reduces students' creativity and critical thinking ability |
|  |  | One can become very dependent on ChatGPT |
|  |  | Other |
|  | If other, please comment. This question only appears if "Other" is selected in question (26). |  |
| 27 | Will ChatGPT replace traditional study methods (e.g., textbooks, lectures) in pharmacy education? How much do you agree with this statement? | Likert Scale § |
| 28 | Artificial intelligence will take over human intelligence in the future. How much do you agree with this statement? | Likert Scale § |

§: Likert Scale, Strongly disagree, disagree, neither agree or disagree, agree, Strongly agree
