# Supplementary material for: Short-term learning effect of ChatGPT on pharmacy students' learning
Source: Explor Res Clin Soc Pharm. 2024 Jul 23;15:100478. doi: 10.1016/j.rcsop.2024.100478 (PMC11321390; doi:10.1016/j.rcsop.2024.100478)
Supplement: Supplementary file 2 — Supplementary material 2 [file mmc2.docx]

## Appendix B: Directed Acyclic Graph for variable selection.


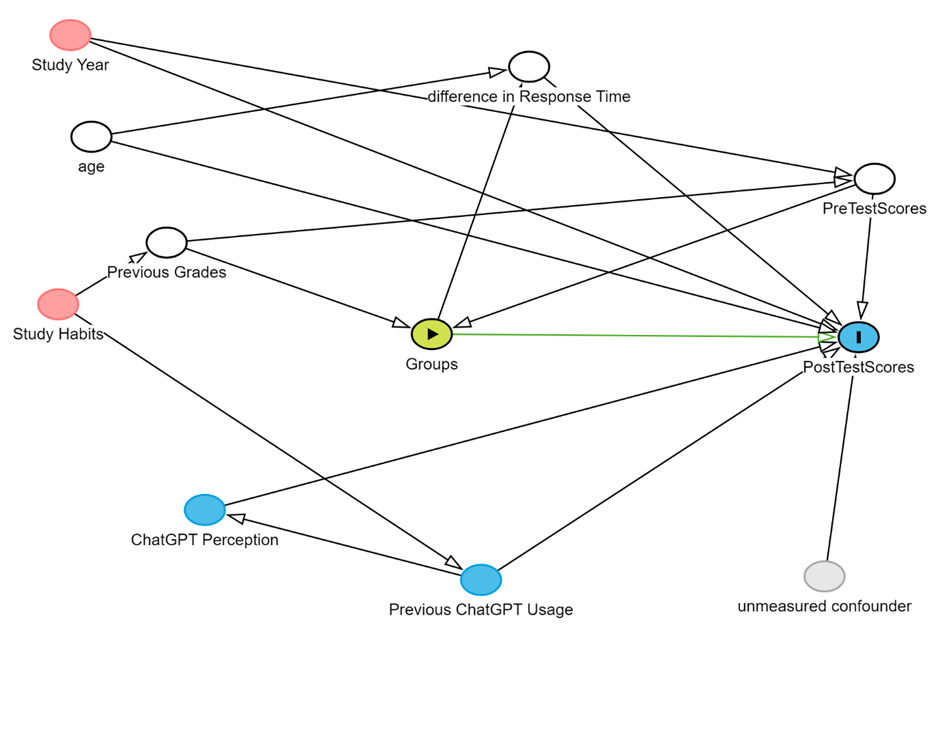


*Figure B1. A Directed Acyclic Graph (DAG) model, the green-colored variable represents the exposure (intervention vs control groups). The blue-colored variables with a dash inside represents the outcome. White-colored variables represent the variables that have been adjusted for to isolate the direct effect of exposure on the outcome.*
