# Supplementary material for: Short-term learning effect of ChatGPT on pharmacy students' learning
Source: Explor Res Clin Soc Pharm. 2024 Jul 23;15:100478. doi: 10.1016/j.rcsop.2024.100478 (PMC11321390; doi:10.1016/j.rcsop.2024.100478)
Supplement: Supplementary file 3 — Supplementary material 3 [file mmc3.docx]

## Appendix C: Linear regression model output

*Table C1 Results of multivariable linear regression of the effect of using ChatGPT (intervention) on score of knowledge test.*

|  |  | Coefficient | 95% Confidence intervall | P-value |
| --- | --- | --- | --- | --- |
| Intervention (using ChatGPT) | | 0.521 | (-1.085, 2.127) | 0.507 |
| Grades | |  |  |  |
|  | A | 0.144 | (-2.897, 3.184) | 0.923 |
|  | B | Reference |  |  |
|  | C | 0.094 | (-1.720, 1.907) | 0.916 |
|  | D | -0.749 | (-3.333, 1.835) | 0.553 |
|  | E | -0.451 | (-4.795, 3.894) | 0.831 |
|  | Not Answered | -0.785 | (-2.884, 1.313) | 0.445 |
| Pre-test score | | 0.677 | (0.263, 1.091) | 0.003 |
| Difference in response time (minutes) | | 0.112 | (-0.062, 0.285) | 0.195 |
| Age | | -0.075 | (-0.207, 0.058) | 0.254 |
| Intercept | | 2.287 | (-1.049, 5.622) | 0.169 |
