# Supplementary material for: Short-term learning effect of ChatGPT on pharmacy students' learning
Source: Explor Res Clin Soc Pharm. 2024 Jul 23;15:100478. doi: 10.1016/j.rcsop.2024.100478 (PMC11321390; doi:10.1016/j.rcsop.2024.100478)
Supplement: Supplementary file 4 — Supplementary material 4 [file mmc4.docx]

## Appendix D: Linear regression assumptions:

We checked for the following linear regression assumptions:

***Linearity and Homoscedasticity***

Residual vs fitted plot (figure 1) was used to determine linearity. The residuals are scattered randomly around the horizontal line at zero with no clear pattern, which suggests linearity.


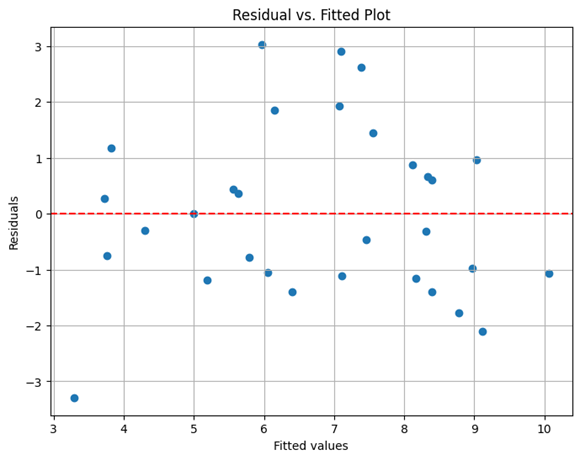


*Figure D1. Residual vs fitted values randomly scattered around zero indication no violation of the linearity assumption.*

***Multicollinearity***

Variance Inflation Factors (VIFs) were calculated for the model predictors. The VIFs measure how much the variance of an estimated coefficient increases if the predictors are correlated. VIF > 5 indicates that multicollinearity may be present; and VIF > 10 indicates a certain multicollinearity among the variables. All values were well below 5 indicating no presence of multicollinearity (range 1.19 to 1.63).

***Independence***

Durbin-Watson Statistic test is calculated to be 1.717 indicating no strong autocorrelation in the residuals and supporting the assumption of independence.

***Normality of model residuals***

A Quantile-Quantile (Q-Q) Plot and Shapiro-Wilk test were used to check for the normality of model residuals. The plot showed that model residuals generally follow the line which indicates the theoretical quantiles of normal distribution suggesting no violation of the normality assumption (Figure 2). Shapiro-Wilk test p-value was 0.601 indicating no significant breach of normality.


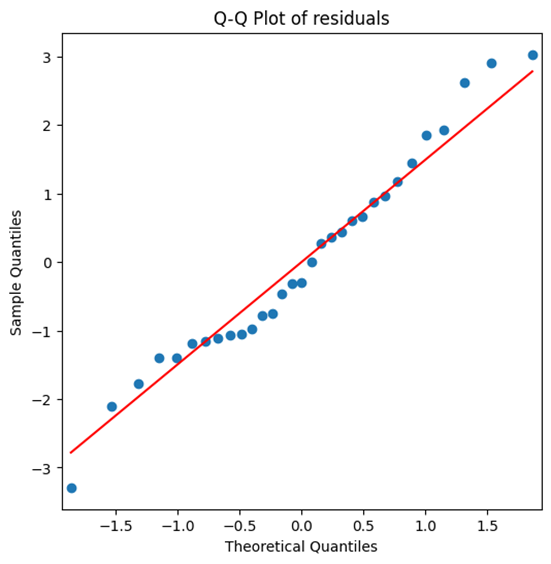


*Figure D2. A Q-Q plot to check for normality. The plot shows that model residuals generally follow the pattern of theoretical normal distribution.*
